# ﻿Three new species of *Trichoderma* (Hypocreales, Hypocreaceae) from soils in China

**DOI:** 10.3897/mycokeys.97.101635

**Published:** 2023-05-02

**Authors:** Rui Zhao, Li-Juan Mao, Chu-Long Zhang

**Affiliations:** 1 Ministry of Agriculture Key Laboratory of Molecular Biology of Crop Pathogens and Insects, Key Laboratory of Biology of Crop Pathogens and Insects of Zhejiang Province, Institute of Biotechnology, Zhejiang University, Hangzhou 310058, China Zhejiang University Hangzhou China; 2 Analysis Center of Agrobiology and Environmental Science, Zhejiang University, Hangzhou, Zhejiang 310058, China Zhejiang University Hangzhou China

**Keywords:** Hypocreales, new species, phylogenetic analysis, taxonomy, *Trichoderma*

## Abstract

*Trichoderma* spp. are diverse fungi with wide distribution. In this study, we report on three new species of *Trichoderma*, namely *T.nigricans*, *T.densissimum* and *T.paradensissimum*, collected from soils in China. Their phylogenetic position of these novel species was determined by analyzing the concatenated sequences of the second largest nuclear RNA polymerase subunit encoding gene (*rpb2*) and the translation elongation factor 1– alpha encoding gene (*tef1*). The results of the phylogenetic analysis showed that each new species formed a distinct clade: *T.nigricans* is a new member of the *Atroviride* Clade, and *T.densissimum* and *T.paradensissimum* belong to the *Harzianum* Clade. A detailed description of the morphology and cultural characteristics of the newly discovered *Trichoderma* species is provided, and these characteristics were compared with those of closely related species to better understand the taxonomic relationships within the *Trichoderma*.

## ﻿Introduction

The genus *Trichoderma* (Ascomycota, Sordariomycetes, Hypocreales) is widely studied and applied because of their economical and ecological significance. In agriculture, they are avirulent plant symbionts used for plant protection and growth promotion (Harman et al. 2004), and as a biological agent to control of fungal diseases (Lorito et al. 2010; Zin and Badaluddin 2020). In addition, *Trichoderma* species have been applied for the production of enzymes and bioactive compounds of industrial utility (Ahamed and Vermette 2008; Sun et al. 2016; Stracquadanio et al. 2020). *Trichoderma* species possessing stress tolerance to different environmental factors hold significant promise for addressing environmental issues such as severe contamination (Kredics et al. 2001; Tripathi et al. 2013). Meanwhile, a few of *Trichoderma* species cause disease in cultivated mushrooms or are reported as causes of serious infections in humans (Kuhls et al. 1999; Savoie and Mata 2003). Members of *Trichoderma* are widely distributed in varied ecosystems, and are frequently found on soil, decaying wood, compost, or other organic matter and as endophytes in plant tissues (Samuels 2006; Zheng et al. 2021).

Traditionally, *Trichoderma* species were identified based on their morphology and growth characteristics (Rifai 1969; Bissett 1984, 1991a, b). However, as the *Trichoderma* species richness has increased, it has been difficult to distinguish them because species in this genus are highly similar in morphology (Bissett et al. 2003; Overton et al. 2006). With the development of molecular biology, more reliable identification is provided as DNA barcoding was introduced to recognize *Trichoderma* (Druzhinina et al. 2006). The most commonly used DNA barcode loci are the internal transcribed spacer (ITS), translation elongation factor 1– alpha encoding gene (*tef1*) and the second largest nuclear RNA polymerase subunit encoding gene (*rpb2*) (Druzhinina et al. 2006; Atanasova et al. 2013; Chaverri et al. 2015; Cai and Druzhinina 2021). The combination of multi–gene (*rpb2* and *tef1*) phylogenetic analysis and phenotypic characteristics is usually applied in the species identification of *Trichoderma* (Chaverri and Samuels 2004; Zhu and Zhuang 2015a, b; Zheng et al. 2021; Cao et al. 2022). Recently, Cai and Druzhinina (2021) have developed an authoritative protocol that provides a standard for the molecular identification of *Trichoderma*. It is based on *rpb2* ≥ 99% and *tef1*≥ 97%, one species can be identified. If the unique sequences do not meet the *rpb2* ≥ 99% or *tef1*≥ 97%, it can be considered a new species. This protocol is advocated for the identification of *Trichoderma* species by the International Subcommission on Taxonomy of *Trichoderma* (https://trichoderma.info/; accessed on 18 Oct 2022).

Fungal diversity is enormous in China (Sun et al. 2012; Lu 2019). Since the first record of *Trichoderma* from China in 1895, many new *Trichoderma* species have been ceaselessly discovered, with most of them isolated from soils, litter, mushrooms and endophytes (Zhang et al. 2005; Yu et al. 2007; Zhang et al. 2007; Li et al. 2013; Zhu and Zhuang 2015a, b; Chen and Zhuang 2016; Qin and Zhuang 2016; Chen and Zhuang 2017; Qiao et al. 2018; Gu et al. 2020; Zhang et al. 2020; Zheng et al. 2021; An et al. 2022; Cao et al. 2022). In a previous study conducted by Dou et al. (2019), a total of 485 Trichoderma strains were obtained from soils in three provinces of China: Shanxi, Shaanxi, Shandong. The online multilocus identification system (MIST) was employed in a previous study conducted by Dou et al. (2020) to re-identify *Trichoderma*. The present study therefore had to identify new taxa, the sequences of which do not meet the known *Trichoderma* species, based on the multi loci phylogenetic analysis and morphological features observation.

## ﻿Materials and methods

### ﻿Isolation of strains

In accordance with a prior study by Dou et al. (2019), a total of 485 Trichoderma strains were extracted from soil samples gathered from three provinces in China. Of these strains, 334 were sourced from Shandong, 107 from Shanxi, and 44 from Shaanxi The isolation of these strains was aided by the use of a selective medium (Dou et al. 2019).

All strains of *Trichoderma* were kept in 4 °C Refrigerator and –80 °C Ultra Low Temperature Refrigerator in the Ministry of Agriculture Key Laboratory of Molecular Biology of Crop Pathogens and Insects, Institute of Biotechnology, Zhejiang University, Hangzhou, China. In addition, the holotype and ex-type culture were deposited in the China General Microbiological Culture Collection Center (CGMCC; https://www.cgmcc.net/english/; accessed on 16 Sep 2022).

### ﻿Morphological characterizations

The morphological observation of the colonies was based on strains grown on potato dextrose agar (PDA; 10g potato extract, 20g dextrose, 13g agar, 1 L distilled water), cornmeal dextrose agar (CMD; 40g cornmeal, 20g dextrose, 15g agar, 1 L distilled water), malt extract agar (MEA; 20g malt extract, 15g agar, 1 L distilled water), and synthetic low nutrient agar (SNA; 1 g KH_2_PO_4_, 1 g KNO_3_, 0.5 g MgSO_4_, 0.5 g KCl, 0.2 g glucose, 0.2 g sucrose, 15 g agar, 1 L distilled water) medium for 7 d in an incubator at 25 °C with alternating 12 h/12h light/dark cycle. Growth–rate trials were performed on 9 cm Petri dishes with CMD, PDA, MEA and SNA at 25 °C, 30 °C, and 35 °C. The Petri dishes were incubated in darkness for up to 1 week or until the colony covered the agar surface. Colony radii were measured daily, and trials were replicated three times.

Microscopic preparations were made by mounted on lactic acid, and at least 30 measurements per structure were documented and examined under a Nikon Eclipse 80i microscope (Nikon Corp.). Length (L) and width (W) of the phialides, conidia and chlamydospores were measured, respectively, and the ratio of length to width was calculated. Measurement values are expressed as (a–)b–c(–d), where (a) represents the lowest extreme value, b–c contains the minimum value of 90% of the calculated values, and (d) denotes the highest extreme value. The letter “n” indicates the total number of measurements taken (Aignon et al. 2021; Li et al. 2021).

### ﻿DNA extraction, polymerase chain reaction (PCR) and sequencing

The mycelia of pure cultures were scraped directly from plates after 2–3 d growth on PDA at 25 °C and used to extract DNA, and the genomic DNA was extracted as described by Jiang et al. (2016). For the amplifications of *rpb2* and *tef1* gene fragments, two different primer pairs were used EF1/EF2 for *tef1* (O’Donnell et al. 1998) and fRPB2–7cR/fRPB2–5F for *rpb2* (Liu et al. 1999). The polymerase chain reaction (PCR) amplifications were performed in a total reaction volume of 20 μL, including 10 μL of Easy Flash PCR MasterMix (Easy–Do, China), 0.8 μL of each primer (10 μM), 0.4 μL genomic DNA (~0.2 μg). PCR reactions were run in a LifePro Thermal Cycler (Technology Co., Ltd. Hangzhou, China) following the PCR thermal cycle programs described by Zhu and Zhuang (2015b). PCR products were purified with the PCR product purification kit and sequencing was carried out in both directions with the same primers on an ABI 3730 XL DNA sequencer (Applied Biosystems, Foster City, CA, USA) by Sunya Biotechnology Co., Hangzhou, China. Sequences generated in this study are deposited in GenBank and the accession numbers are provided in Table 1.

**Table 1.** Strain numbers and corresponding GenBank accession numbers of sequences used for phylogenetic analyses.

Species name	Strain number	GenBank accession numbers	
		*rpb2*	*tef1*
*T.afroharzianum*	CBS 124620 ^ET^	FJ442691	FJ463301
*T.afroharzianum*	GJS 04–193	FJ442709	FJ463298
*T.anaharzianum*	YMF 1.00383 ^T^	MH158995	MH183182
*T.asiaticum*	YMF 1.00168	MH262575	MH236492
*T.asiaticum*	YMF 1.00352 ^T^	MH158994	MH183183
*T.atrobrunneum*	CBS 548.92 ^T^	–	AF443942
*T.atrobrunneum*	GIS 04–67	FJ442724	FJ463360
*T.atrobrunneum*	GJS 05–101	FJ442745	FJ463392
*T.atroviride*	CBS 119499	FJ860518	FJ860611
*T.atroviride*	CBS 142.95 ^ET^	EU341801	AY376051
*T.breve*	CGMCC 3.18398 ^T^	KY687983	KY688045
*T.breve*	HMAS 248845	KY687984	KY688046
***T.densissimum***	**T31818**	**OP357965**	**OP357967**
***T.densissimum***	**T32434 = CGMCC 3.24126 ^T^**	**OP357966**	**OP357971**
***T.densissimum***	**T32465**	**OP357963**	**OP357972**
***T.densissimum***	**T32353**	**OP357964**	**OP357970**
*T.guizhouense*	CBS 131803 ^T^	JQ901400	JN215484
*T.guizhouense*	HGUP 0039	JQ901401	JX089585
*T.harzianum*	CBS 226.95 ^ET^	AF545549	AF348101
*T.harzianum*	TRS55	KP009121	KP008803
*T.harzianum*	TRS94	KP009120	KP008802
***T.nigricans***	**T32450**	**OP357958**	**OP357973**
***T.nigricans***	**T32794**	**OP357960**	**OP357975**
***T.nigricans***	**T32781 = CGMCC40314 ^T^**	**OP357959**	**OP357974**
*T.obovatum*	YMF 1.06211 ^T^	MT038432	MT070144
*T.obovatum*	YMF 1.6190	MT038433	MT070143
***T.paradensissimum***	**T31823 = CGMCC 3.24125 ^T^**	**OP357962**	**OP357968**
***T.paradensissimum***	**T31824**	**OP357961**	**OP357969**
*T.paratroviride*	CBS 136489 ^T^	KJ665321	KJ665627
*T.paratroviride*	S489	KJ665322	KJ665628
*T.paraviride*	YMF 1.04628 ^T^	MK775513	MK775508
*T.pholiotae*	JZBQH12 ^T^	ON649972	ON649919
*T.pholiotae*	JZBQH11	ON649971	ON649918
*T.pyramidale*	CBS 135574 ^ET^	KJ665334	KJ665699
*T.pyramidale*	T20	KX632570	KX632627
*T.simile*	YMF 1.06201 ^T^	MT052184	MT070154
*T.simile*	YMF1.6180	MT052185	MT070153
*T.uncinatum*	YMF 1.04622 ^T^	MK795990	MK795986
*T.viride*	TRS575	KP009081	KP008931
*T.viride*	CBS 119325 ^ET^	EU711362	DQ672615
*T.zelobreve*	CGMCC 3.19695 ^T^	MN605872	MN605883
*T.zelobreve*	CGMCC 3.19696	MN605873	MN605884
*T.zeloharzianum*	YMF 1.00268 ^ET^	MH158996	MH183181
*Protocreafarinosa*	CBS 121551 ^T^	OP357962	EU703889
*Protocreapallida*	CBS 299.78 ^ET^	EU703948	EU703900

Note: Newly–sequenced material is indicated in bold type. T Indicates a type culture. ET Indicates an epitype culture.

### ﻿Phylogenetic analyses

The phylogeny was constructed with the concatenated sequences of *rpb2* and *tef1*. The species closely related to our strain were determined by NCBI BLAST searches with *rpb2* and *tef1* sequences (Altschul et al. 1990; https://blast.ncbi.nlm.nih.gov/Blast.cgi/; accessed on 16 Jun 2022), and the closely related sequences were retrieved from NCBI database for subsequent phylogenetic analysis. The GenBank accession numbers of sequences retrieved are provided in Table 1. The sequences were aligned with MAFFT (Katoh and Standley 2013), and then the alignments were manually adjusted with MEGA7 (Kumar et al. 2018) and the fragments that were suitable for molecular identification were trimmed according to Cai and Druzhinina (2021). The trimmed sequences were concatenated using SequenceMatrix v.1.8 (Vaidya et al. 2011). The following phylogenetic analysis was performed in PhyloSuite platform (Zhang et al. 2020). The best–fit partition model was selected using ModelFinder (Kalyaanamoorthy et al. 2017) according to BIC criterion. Maximum likelihood (ML) phylogenies were inferred using IQ–TREE (Lam–Tung et al. 2015) under Edge–linked partition model for 5000 ultrafast (Minh et al. 2013) bootstraps, as well as the Shimodaira–Hasegawa–like approximate likelihood–ratio test (Guindon et al. 2010). Bayesian Inference phylogenies were inferred using MrBayes 3.2.6 (Ronquist et al. 2012) under partition model. The phylogenetic tree was visualized in FigTree v1.4.3. (http:/tree.bio.ed.ac.uk/software/figtree/; accessed on 04 Oct 2016) with maximum likelihood bootstrap proportions (MLBP) greater than 70% and Bayesian inference posterior probabilities (BIPP) greater than 0.9, as shown at the nodes.

## ﻿Results

### ﻿Sequence analysis

The comparison of *rpb2* and *tef1* sequences between the query strain and the reference strain revealed that the similarity did not meet the rpb2 ≥ 99% and tef1 ≥ 97% criteria as outlined in Table 2. Additionally, the query strain exhibited unique *tef1* and *rpb2* sequences that do not conform to the sp∃!(*rpb2*_99_≅*tef1*_97_) standard for known *Trichoderma* species, according to Cai and Druzhinina (2021). These findings suggest that these strains could potentially be classified as new species, and therefore, phylogenetic analyses were conducted on their *rpb2* and *tef1* sequences.

**Table 2.** The similarity of *rpb2* and *tef1* between the query species and related species.

Query species	Related species	Sequences similarity value(%)	
		*rpb2*	*tef1*
*Trichodermanigricans* T32781^T^	*T.atroviride* CBS 142.95 ^ET^	97.91	91.29
	*T.obovatum* YMF 1.06211 ^T^	98.15	86.68
	*T.paratroviride* CBS 136489 ^T^	98.65	87.53
	*T.uncinatum* YMF 1.04622^T^	98.56	94.40
*T.paradensissimum* T31818^T^	*T.densissimum* T31823^T^	97.54	99.20
	*T.asiaticum* YMF1.00352^T^	96.92	98.06
	*T.guizhouense* HGUP 0038 ^T^	97.05	98.29
	*T.pholiotae* JZBQH12^T^	97.42	99.16
	*T.simile* YMF 1.06201^T^	97.17	97.83
*T.densissimum* T31823^T^	*T.paradensissimum* T31818^T^	97.54	99.20
	*T.asiaticum* YMF 1.00352^T^	97.79	98.06
	*T.guizhouense* HGUP 0038 ^T^	97.17	98.29
	*T.pholiotae* JZBQH12^T^	98.04	100
	*T.simile* YMF 1.06201^T^	97.66	97.83

Note: T Indicates a type culture. ET Indicates an epitype culture.

### ﻿Multi-locus phylogeny

**Figure 1.**
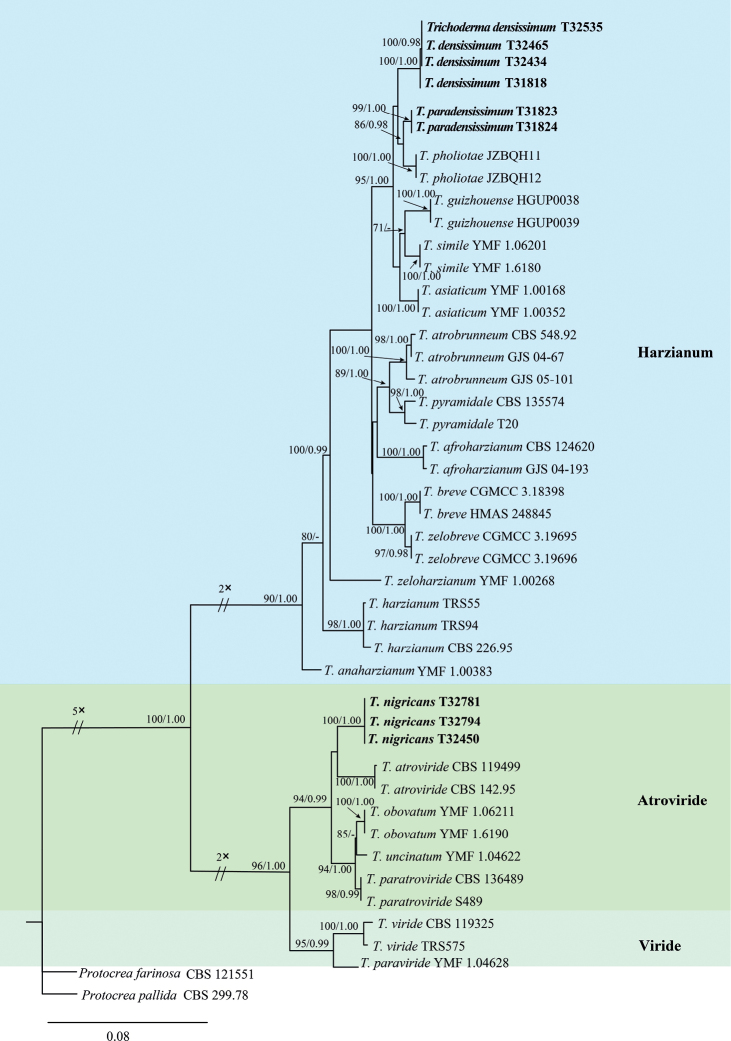
Phylogenic tree generated by the maximum likelihood analysis using the concatenated sequences of *rpb*2 and *tef1* loci of the genus *Trichoderma*. Maximum Likelihood Bootstrap values ≥70% (left) and Bayesian posterior probability values ≥0.9 (right) are indicated at nodes (MLBP/BIBP). *Protocreafarinosa* CBS 121551 and *P.pallida* CBS 299.78 were chosen as the outgroup. Novel species proposed here are indicated in bold.

Multi-loci phylogenetic analyses were performed on sequences obtained from 43 strains, consisting of 30 strains from the *Harzianum* Clade, 10 strains from the *Atroviride* Clade, and 3 strains from the *Viride* Clade. The combined *rpb2* and *tef1* regions were further analyzed by the methods of ML and BI, with *Protocreafarinosa* CBS 121551 and *P.pallida* CBS 299.78 as the outgroup. The tree topology derived from the ML analysis (Fig. 1) was consistent with that obtained in a BI analysis. However, details regarding the BI analysis were not provided in the text. All strains formed a monophyletic group with higher statistical support, designated as *T.nigricans* (MLBP/BIBP = 100/1.00), *T.densissimum* (MLBP/BIBP = 100/1.00) and *T.paradensissimum* (MLBP/BIBP = 99/1.00). Of the three new species, *T.nigricans* belonged to the *Atroviride* Clade, whereas *T.densissimum* and *T.paradensissimum* were located in the *Harzianum* Clade (Fig. 1). *Trichodermanigricans* was closely related with *T.atroviride*, and associated with *T.obovatum*, *T.uncinatum*, and *T.paratroviride*. This clade had high statistics support (MLBP/BIBP = 94/0.99). *Trichodermadensissimum* was closely related with *T.paradensissimum*, and associated with *T.pholiotae*, *T.guizhouense*, *T.asiaticum* and *T.simile*, with high support value (MLBP/BIBP = 95/1.00).

## ﻿Taxonomy

### ﻿ Trichoderma nigricans

Taxon classificationFungiHypocrealesHypocreaceae

C.L. Zhang sp. nov.

DAB7A9EF-FBD5-5F48-A3DF-958ED1242E05

 845506

Fig. 2

#### Etymology.

The Latin specific epithet “*nigricans*” refers to the “blackish green” color of the mass of conidia.

#### Diagnosis.

Phylogenetically, *T.nigricans* was found to form a distinct clade and was closely related to *T.atroviride*, *T.paratroviride*, *T.obovatum*, and *T.uncinatum* (Fig. 1). In terms of growth characteristics, *T.nigricans* was observed to have a larger colony radius on CMD after 72 h, and its mycelium covered the plate at both 25 °C and 30 °C. On PDA, *T.nigricans* grew faster than *T.atroviride*, *T.paratroviride*, *T.obovatum*, and *T.uncinatum* at 25 °C, with its mycelium also covering the plate.

**Figure 2.**
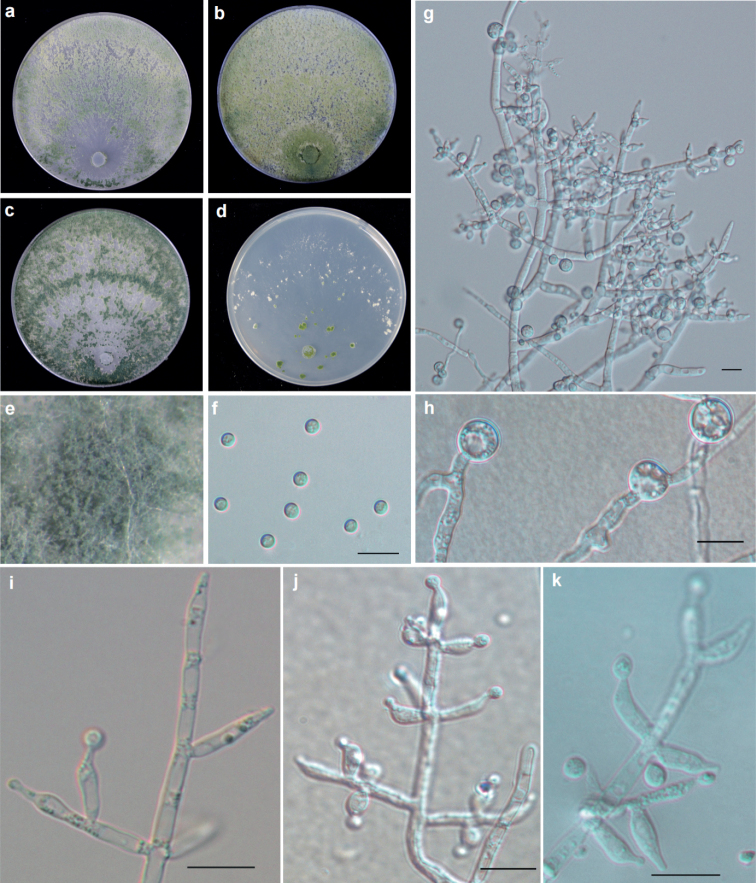
Cultures and anamorph of *T.nigricans* strain T32781 **a–d** cultures on different media at 25 °C with a 12 h light and 12 h darkness cycle after 7 d (**a** on PDA**b** on MEA**c** on CMD**d** on SNA) **e** Conidiation pustules on PDA after 7 d **f** conidia **g, i–k** conidiophores and phialides (**g, k** on CMD 3d **i** on PAD 3d **j** on SNA 3d,) **h** chlamydospores. Scale bars: 10 μm (**f–k**).

#### Type.

China: Shandong Province, Dezhou City, 37°21'07"N, 116°23'40"E, 5 m alt., isolated from soils of peach rhizosphere. Oct 2015, Y. Jiang T32781 (Holotype CGMCC 40314, stored in a metabolically inactive state. Ex-type culture CGMCC 40314).

#### Description.

Optimal growth at 25 °C, slow at 35 °C on all media.

Colony radius on CMD after 72 h: mycelium covers the plate at 25 °C and 30 °C, 20–22 mm at 35 °C. Colony well–defined, hyaline, sparse aerial mycelia, indistinctly zonate, conidiation begins to develop within 72 h, white at first and turning green after 3–4 d. After 7 d, abundant dark green conidiation around the margin, radially arranged within 2–3 ill–defined concentric zones in the outer half of the colony. Abundant chlamydospores. No diffusing pigment noted, pleasant odor apparent.

Colony radius on PDA after 72 h: mycelium covers the plate at 25 °C, 55–61 mm at 30 °C, 16 mm at 35 °C. Colony similar to CMD but growth a little slower, colony not dark green. Colony well–defined at 35 °C, abundant white thick aerial mycelia. Chlamydospores abundant. No diffusing pigment noted, obvious pleasant odor.

Colony radius on MEA after 72 h: 58–60 mm at 25 °C, 53–55 mm at 30 °C, 11–12 mm at 35 °C. Colony also similar to CMD, but conidiation is yellow green, more abundant around the inoculation plug, uniform distribution all around. No diffusing pigment noted, odor indistinct.

Colony radius on SNA after 72 h: 5–7 mm at 25 °C, 5–6 mm at 30 °C and 35 °C. Colonies well–defined, hyaline, scant aerial mycelia. Slight conidiation dispersedly distributed around the inoculation plug, with white floccose indistinctly zonate tufts or pustules in the margin. No diffusing pigment noted, odor indistinct. Conidiophores consisting of a main axis with side branches mostly at right angles or slightly inclined upward; branches straight or curved, often only longer in basal positions, not re–branching, solitary, paired or in whorls of three. Phialides solitary or commonly in whorls of 2–3, variable in shape, either narrowly lageniform to subulate, particularly when terminal on the main axis, or stout to nearly ampulliform and distinctly swollen, sometimes ampulliform to subglobose, (4.7–)6.0–8.9(–12.1) × (2.5–)2.9–3.4(–4.5) μm (mean =7.7 × 3.3 μm), base (1.5–)1.6–2.6(–3.0) μm (mean = 2.1 μm); phialide length/width ratio (1.2–)1.8–2.9(–3.6) (mean = 2.4) (n = 30). Conidia subglobose to globose, green, smooth, (3.0–)3.2–3.6(–3.9) ×(2.8–)3.1–3.4(–3.8) μm (mean = 3.3×3.4 μm) with length/width ratio of 1.0–1.1 (mean = 1.1) (n = 30). Abundant chlamydospores, common single, sometimes terminal and intercalary, globose to subglobose, (7.2–)7.8–9.2(–10.1) × (6.1–)7.1–9.0(–9.7) μm (mean = 8.6×8.1 μm) (n = 30).

#### Sexual morph.

Unknown.

#### Substrate.

Soil.

#### Distribution.

China, Shandong Provinces.

#### Additional material examined.

China: Shandong Province, Jinan City, 36°33'45"N, 116°57'05"E, 105 m alt., isolated from corn soils. Aug 2015, Y. Jiang T32450. China: Shandong Province, Dezhou City, 37°21'07"N, 116°23'40"E, 5 m alt., isolated from soils of corn rhizosphere, Oct 2015, Y. Jiang, T32794.

#### Notes.

*Trichodermanigricans* can be distinguished from similar species based on growth. After 72 h at 25 °C, *T.nigricans* mycelium covers the plate on PDA and CMD, *T.atroviride* grows to 42.8–60.5 mm on PDA, *T.obovatum* grows to 38–41 mm on CMD, *T.uncinatum* grows to 55–62 mm on CMD, *T.paratroviride* to 49–62 mm on CMD and 54–56 mm on PDA (Samuels et al. 2002; Jaklitsch and Voglmayr 2015; Zheng et al. 2021). In addition, it can be distinguished by its chlamydospores and odor. At 35 °C the growth of *T.nigricans* is restricted, and no growth occurs in *T.paratroviride* and *T.uncinatum*. Chlamydospores are either unobserved or uncommon in *T.obovatum*, *T.uncinatum*, and *T.paratroviride*. Meanwhile, the chlamydospores of *T.atroviride* and *T.nigricans* are abundant, and the volume in *T.atroviride* is usually larger than those in *T.nigricans* [(5.2–)8.5–12.0(–16.3) vs. (7.2–)7.8–9.2(–10.1) × (6.1–)7.1–9.0(–9.7) μm]. On PDA, the odor of *T.paratroviride* is pungent; it is indistinct in *T.obovatum* and *T.uncinatum*, and pleasant in *T.atroviride* and *T.nigricans*.

### ﻿ Trichoderma densissimum

Taxon classificationFungiHypocrealesHypocreaceae

C.L. Zhang sp. nov.

4B2C5C73-F952-50D8-880A-36015FD1AF51

 845507

Fig. 3

#### Etymology.

The Latin specific epithet “*densissimum*” refers to the thick wall of chlamydospores of this species.

#### Diagnosis.

It is easily distinguished from these related species by its relatively large chlamydospores (11.7–)13.3–16.4 (–19.5) × (11.5–)12.8–14.6–12.8 (–16.0) μm (mean = 14.8 × 13.6 μm) (n = 30).

**Figure 3.**
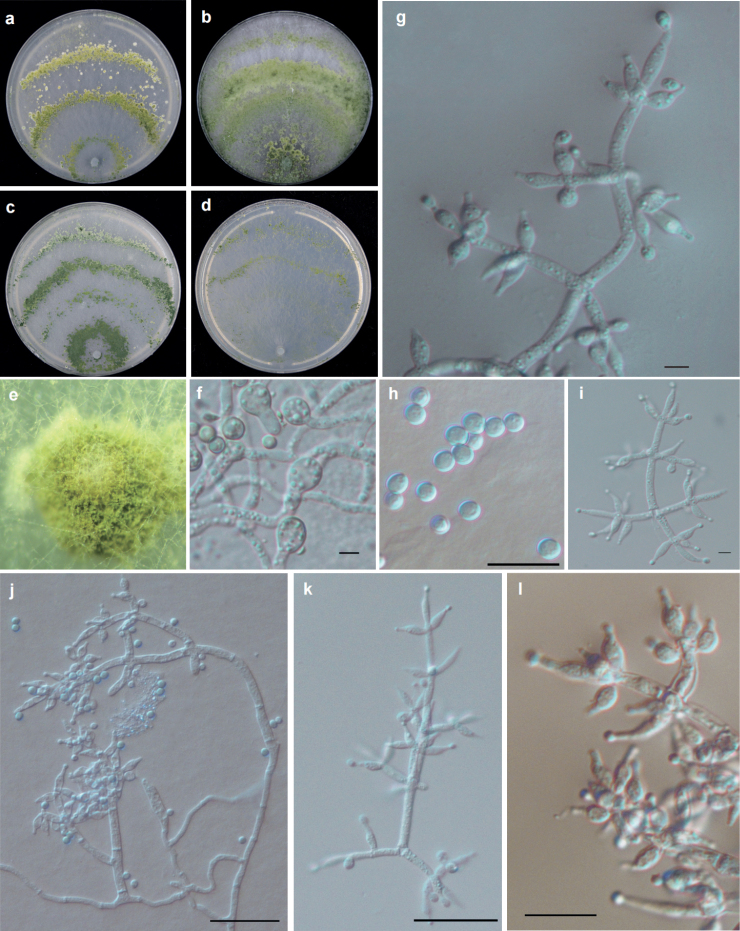
Cultures and anamorph of *T.densissimum* strain T32434 **a–d** cultures on different media at 25 °C with a 12 h light and 12 h darkness cycle after 7 d (**a** on PDA**b** on MEA**c** on CMD**d** on SNA) **e** conidiation pustules on PDA after 7d **g, i–l** conidiophores and phialides (**g, i–k** on CMD 3d **l** on SNA 3d) **f** chlamydospores **h** conidia. Scale bars: 10 μm (**f–l**).

#### Type.

China: Shandong Province, Weifang City, 36°38'27"N, 119°01'21"E, 80 m alt., isolated from soils of apple tree rhizosphere. Oct 2015, Y. Jiang T32434 (Holotype CGMCC 3.24126, stored in a metabolically inactive state. Ex-type culture CGMCC 3.24126).

#### Description.

Optimum temperature for growth is 30 °C on CMD, MEA and SNA and 25 °C on PDA. Growth slow at 35 °C on PDA and SNA. Chlamydospores are common on all media.

Colony radius on CMD after 72 h: 38–45 mm at 25 °C, 55–62 mm at 30 °C, 42–43 mm at 35 °C. Colonies well–defined, white, thin, aerial hyphae sparse. Conidiation was noted after 2 d around the inoculation plug, which was white at first, turning yellow green after 3–4 d, then dark green after 5–6 d. Conidiation formed 4 obvious concentric zones. No diffusing pigment noted, odor indistinct. Chlamydospores common single, sometimes terminal and intercalary, globose to subglobose, (11.7–)13.3–16.4(–19.5) × (11.5–)12.8–14.6–12.8(–16.0) μm (mean = 14.8 × 13.6μm); with length/width ratio of 1.0 × 1.3 (mean = 1.1) (n = 30).

Colony radius on PDA after 72 h: 61–66 mm at 25 °C, 60–63 mm at 30 °C, 24–31 mm at 35 °C. Colony white, regularly circular, distinctly zonate; mycelium dense and radial. Conidiation in the form on pustules, yellow–green, relatively abundant in the zonation regions. No diffusing pigment noted, odor indistinct.

Colony radius on MEA after 72 h: 62–63 mm at 25 °C, 66–67 mm at 30 °C, 44–47 mm at 35 °C. Colonies similar to that on PDA, but indistinctly zonate. No diffusing pigment noted, odor indistinct.

Colony radius on SNA after 72 h: 53 mm at 25 °C, 41–47 mm at 30 °C, 27–32 mm at 35 °C. Colony white; aerial mycelia scant and loose. Conidiation in the form of minute pustules, radial and inconspicuously zonate. No diffusing pigment noted, odor indistinct. Conidiophores pyramidal with opposing branches, the main axis with side branches is sometimes at right angles or inclined upward. The main axis and each branch commonly terminating verticillate, whorl of 3–4 phialides, sometimes in a cruciate whorl, sometimes solitary phialides. Phialides commonly ampulliform, sometimes ampulliform to subglobose (3.4–)5.7–8.0(–10.1) × (1.9–)2.5–2.9(–3.2) μm (mean = 6.2 × 2.6μm), base (1.0–)1.4–2.1(–2.6) μm (mean = 2.2 μm); phialide length/width ratio (1.4–)2.1–3.2(–3.9)(mean = 2.6) (n = 30). Conidia subglobose to globose, green, (2.3–)2.8–3.1(–3.4) × (2.2–)2.4–2.9(–3.3) μm (mean = 2.9 × 2.7 μm), with length/width ratio of 1.0–1.4 (mean = 1.1) (n = 30).

#### Sexual morph.

Unknown.

#### Substrate.

Soil.

#### Distribution.

China, Shandong and Shanxi provinces.

#### Additional material examined.

China: Shandong Province, Jinan City, 36°32’33”N, 117°01’08”E, 201 m alt., isolated from soils of wheat, Jun 2015, Y. Jiang (T31818); Shandong Province, Jining city, 34°56’21”N, 116°29’03”E, 34 m alt., isolated from soils of peach, Aug 2015, Y. Jiang T32353; Shaanxi Province, Baoji city, 34°23’25”N, 107°10’18”E, 802 m alt., isolated from soils of corn, Aug 2015, Y. Jiang T32465.

#### Notes.

Although *T.densissimum*, *T.paradensissimum* and *T.guizhouense* share similar conidia and pyramidal conidiophores, *T.densissimum* cannot produce pigments while *T.paradensissimum* and *T.pholiotae* can produce yellowish pigment on PDA and CMD at 35 °C in the dark (Li et al. 2013; Cao et al. 2022). Characterized by producing globose to subglobose chlamydospores, the chlamydospores of *T.simile* are elliptic or round, unobserved in *T.guizhouense* and *T.asiaticum* (Jaklitsch and Voglmayr 2015; Zheng et al. 2021).

### ﻿ Trichoderma paradensissimum

Taxon classificationFungiHypocrealesHypocreaceae

C.L. Zhang sp. nov.

B5993F20-A067-5EED-B97F-73FC67063C8E

 845508

Fig. 4

#### Etymology.

The Latin specific epithet “*para*” means similar, and “*paradensissimum*” refers to the phylogenetic proximity and morphological similarity to *T.densissimum*.

#### Diagnosis.

*T.paradensissimum* is characterized by the green to yellow and white pustules formed inconspicuously zonate on PDA or MEA at 25 °C of a 12– h photoperiod after 7 d.

**Figure 4.**
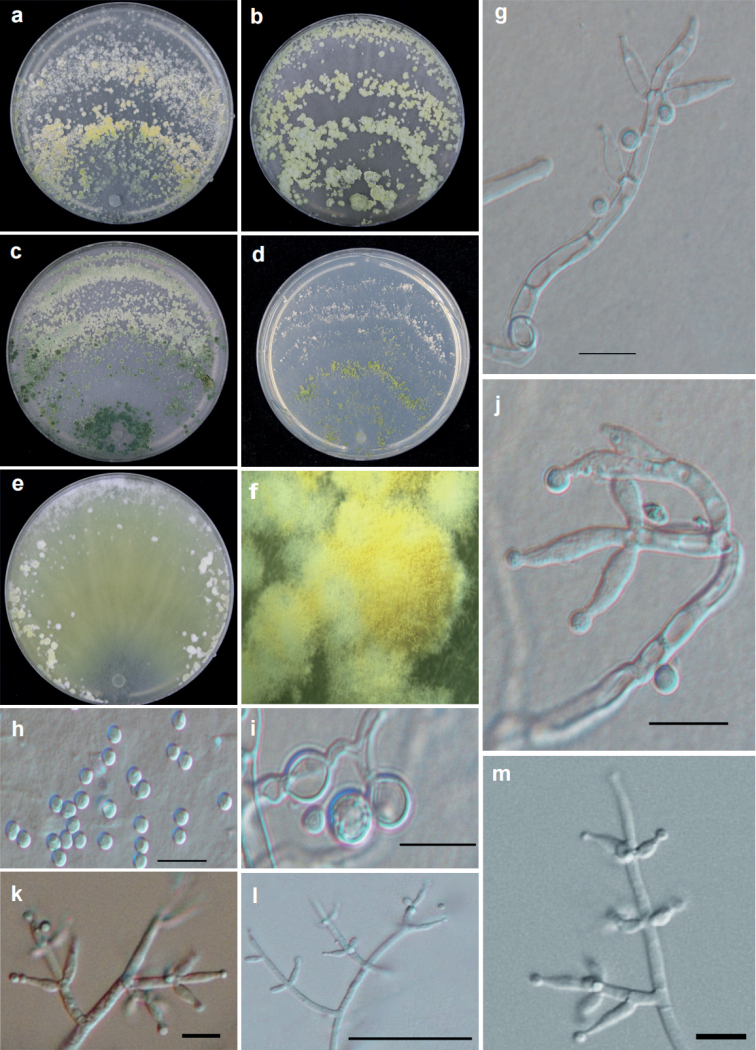
Cultures and anamorph of *T.paradensissimum* strain T31823 **a–d** cultures on different media at 25 °C with a12 h light and 12 h darkness cycle after 7 d (**a** on PDA**b** on MEA**c** on CMD**d** on SNA) **e** culture on PDA at 35 °C with darkness after 7 d **f** conidiation pustules on PDA after 7 d **g, j–m** conidiophores and phialides (**g, j** on CMD 3d **k–m** on SNA 3d) **h** conidia **i** chlamydospores. Scale bars: 10 μm (**g–m**).

#### Type.

China: Shanxi Province, Jincheng City, 35°26'57.9"N, 112°45'19.0"E, 929 m alt., isolated from soils of wheat rhizosphere, Jun 2015, Y. Jiang T31823 (Holotype CGMCC 3.24125, stored in a metabolically inactive state. Ex-type culture CGMCC 3.24125).

#### Description.

Optimum temperature for growth is 30 °C on CMD, PDA and SNA and 25 °C on MEA. Chlamydospores were common on all media.

Colony radius on CMD after 72 h: 40–42 mm at 25 °C, 63–64 mm at 30 °C, 38–40 mm at 35 °C. Colony well–defined, white, aerial myceli loose and radial. White minute pustules were noted after 2 d around the inoculation plug, white at first, turning yellow green after 3–4 d, then dark green after 5–6 d. Around the point of inoculation, conidiation from dark green to pale green, inconspicuously zonate. Distinctive odor absent. The production of pigment was related to light, media and temperature: around the point of inoculation, it was yellowish at 35 °C in the dark.

Colony radius on PDA after 72 h: 59–65 mm at 25 °C, 64–67 mm at 30 °C, 20–24 mm at 35 °C. Colonies similar to that on MEA. Pustules were noted after 4–5 d. After 7 d, the green to yellow and white pustules were formed as inconspicuously zonate. Distinctive odor absent. The production of pigment was related to light and temperature; it was yellowish at 35 °C in the dark.

Colony radius on MEA after 72 h 58–59 mm at 25 °C, 51–53 mm at 30 °C, 34–35 mm at 35 °C. Colonies white and thick, regularly circular and radial, aerial myceli dense. A few white–yellow large pustules formed inconspicuously zonate. Diffusing pigment or distinctive odor absent.

Colony radius on SNA after 72 h 35–37 mm at 25 °C, 43–44 mm at 30 °C, 15–16 mm at 35 °C. Colony pale white; aerial myceli loose. Conidiation was minute pustules, radial and inconspicuously zonate. Around the point of inoculation, the pustules were green, but white far away from the inoculation. Diffusing pigment or distinctive odor absent. Conidiophores pyramidal; the main axis with side branches sometimes at right angles or inclined upward. The main axis and each branch commonly terminating verticillate, whorl of 3 phialides, sometimes solitary. Phialides ampulliform, (5.4–)7.4–11.0(–15.0) × (2.1–)2.7–3.1(–3.3) μm (mean = 9.4 × 2.9 μm), base (1.6–)1.8–2.3(–2.6) μm (mean = 2.0 μm); phialide length/width ratio (2.1–)2.6–3.7(–4.9)(mean = 3.2) (n = 30). Conidia subglobose to globose, green, (2.6–)2.7–3.0(–3.5) × (2.4–)2.5–2.9(–3.2) μm (mean = 2.9 × 2.7 μm), with length/width ratio of 1.0–1.2 (mean = 1.1) (n = 30). Chlamydospores abundant, common single, sometimes terminal and intercalary, globose to subglobose, (4.6–)5.1–6.2(–6.8) × (3.7–)4.6–5.9(–6.7) μm (mean = 5.7 × 5.4 μm); length/width ratio 1.0×1.3(mean = 1.1) (n = 30).

#### Sexual morph.

Unknown.

#### Substrate.

Soil.

#### Distribution.

China, Shanxi Province.

#### Additional material examined.

China: Shanxi Province, Jincheng City, 35°26'58.1"N, 112°45'19.4"E, 929 m alt., isolated from soil of wheat rhizosphere, Jun 2015, Y. Jiang T31824.

#### Notes.

Similar species can be distinguished according to the pigment: *T.paradensissimum* can produce yellowish pigment on PDA and CMD at 35 °C in the dark, whereas *T.guizhouense* typically at 35 °C reverse forming a dull orange to brown pigment. However, *T.densissimum*, *T.asiaticum*, *T.simile* and *T.zelobreve* cannot produce diffusing pigment on PDA. *Trichodermapholiotae* and *T.paradensissimum* can both produce yellow pigment on PDA, but *T.pholiotae* has a slightly fruity odor on both PDA and CMD, while *T.paradensissimum* does not have a distinctive odor (Cao et al. 2022).

## ﻿Discussion

All three new species were isolated from soils. Based on morphology and phylogenetic analyses, the taxonomic positions of three new species were explored. Of these species, *T.nigricans* was grouped into the *Atroviride* Clade, while *T.densissimum* and *T.paradensissimum* were associated with the *Harzianum* Clade.

The genus *Trichoderma* contains at least eight infrageneric clades, of which the *Harzianum* clade is one of the largest (Cai and Druzhinina 2021). The *Harzianum* clade consists of more than 95 accepted species, which are morphologically heterogeneous and phylogenetically complicated (Cao et al. 2022). Two of the newly described species, *T.densissimum* and *T.paradensissimum*, belong to the *Harzianum* Clade, which are closely related to *T.pholiotae*, associated with *T.guizhouense*, *T.asiaticum*, and *T.simile*. The chlamydospores of the *Harzianum* Clade members are usually either rarely numerous or not observed, and this is consistent with observations for *T.guizhouense*, *T.asiaticum*, *T.breve*, *T.bannaense*, and *T.atrobrunneum*, among others. In *T.simile*, the chlamydospores are either elliptic or round in shape (Li et al. 2013; Chaverri et al. 2015; Jang et al. 2018; Gu et al. 2020). In contrast, the chlamydospores of *T.densissimum* and *T.paradensissimum* are numerous, globose to subglobose, and relatively large, especially in *T.densissimum*. Our phylogenetic analyses revealed that *T.densissimum* and *T.paradensissimum* are closely related due to the minimal genetic variation observed in their ITS and *tef1* sequences. Moreover, both species exhibit similar growth characteristics and possess numerous chlamydospores. However, their genetic variation in the sequences of *rpb2* (similarity < 99%) differentiate them as distinct species. In addition, *T.densissimum* exhibits green conidiation with 3–4 distinct concentric zones and no diffusing pigment, while *T.paradensissimum* exhibits inconspicuously zonate green to yellow conidiation with white pustules and yellowish pigment.

*Trichodermaatroviride* and *T.paratroviride* were classified to the *Viride* Clade (Jaklitsch and Voglmayr 2015). However, with the addition of *T.obovatum* and *T.uncinatum*, they were assigned to the *Atroviride* Clade by (Zheng et al. 2021). In this study, the new species *T.nigricans* was also identified as a member of the *Atroviride* Clade. The results of the phylogenetic analysis indicated a close relationship between *T.nigricans* and *T.atroviride*. Morphologically, *T.nigricans* shares many similarities with *T.atroviride*, including the production of a strong coconut odor in PDA cultures and the presence of abundant chlamydospores. *Trichodermanigricans* exhibits a faster growth rate on PDA in comparison to *T.atroviride*, with the former’s mycelium covering a larger area of the plate and its colony radius measuring between 42.8–60.5 mm after 72 h at 25 °C. Colony radius is *T.nigricans* 16 mm vs. *T.atroviride* (0~)0.3~3.2(~8.3) mm at 35 °C (Samuels et al. 2002).

Numerous biological control agents have been derived from species in the *Atroviride* and *Harzianum* clade to effectively control soil–borne diseases (Chaverri et al. 2015), such as *T.atroviride*, *T.guizhouense*, *T.afroharzianum*, and *T.atrobrunneum* (Longa et al. 2010; Rees et al. 2022; Zhang et al. 2022; Zhao et al. 2022). The discovery of *T.nigricans*, *T.densissimum*, and *T.paradensissimum* in this study highlights the diversity of *Trichoderma* in China and provides valuable information for the development of *Trichoderma*-based biocontrol agents. Further research is necessary to explore the diversity of *Trichoderma* in China and to investigate their potential as biocontrol agents against plant diseases.

## Supplementary Material

XML Treatment for Trichoderma nigricans

XML Treatment for Trichoderma densissimum

XML Treatment for Trichoderma paradensissimum
